# A sensitive indicator for the severity of COVID-19: thiol

**DOI:** 10.3906/sag-2011-139

**Published:** 2021-06-28

**Authors:** Özcan EREL, Salim NEŞELİOĞLU, Merve ERGİN TUNÇAY, Esra FIRAT OĞUZ, Funda EREN, Meryem Sena AKKUŞ, Rahmet GÜNER, İhsan ATEŞ

**Affiliations:** 1 Department of Biochemistry, Faculty of Medicine, Yıldırım Beyazıt University, Ankara, Turkey; 2 Central Biochemistry Laboratory, Ankara City Hospital, Ankara Turkey; 3 Central Research Laboratory, Yıldırım Beyazıt University, Ankara Turkey; 4 Department of Infectious Diseases and Clinical Microbiology, Faculty of Medicine, Yıldırım Beyazıt University, Ankara Turkey; 5 Department of Infectious Diseases and Clinical Microbiology, Ankara City Hospital, Ankara Turkey; 6 Department of Internal Medicine, Ankara City Hospital, Ankara Turkey

**Keywords:** COVID-19, inflammation, immune response, severity, thiol

## Abstract

**Background/aim:**

Thiol status is a good reflector of the cellular redox and have vital roles in various cellular signaling pathways. The purpose of the study was to investigate thiol status in patients with SARS-CoV-2 infection.

**Materials and methods:**

A total of 587 subjects (517 patients/70 healthy controls) were enrolled in the study.The patients were categorized into the groups regarding to the severity of disease (mild, moderate, severe, and critical).Thiol status of all groups were compared.

**Results:**

The patients had significantly diminished thiol levels compared to controls. Thiol levels were gradually decreased as the severity of the disease increased. Logistic regression analyses identified that thiol concentrations were an independent risk factor for the disease severity in each phase (mild group OR 0.975, 95%CI 0.965-0.986; moderate group, OR 0.964, 95%CI 0.953-0.976; severe group OR 0.953, 95%CI 0.941-0.965; critical group OR 0.947, 95%CI 0.935-0.960).Thiol test exhibited the largest area under the curve at 0.949, with the highest sensitivity (98.6%) and specificity (80.4%).

**Conclusions:**

Depleted thiol status was observed in SARS-CoV-2 infection. Decline of the thiol levels by degrees while the severity of infection increased was closely related to the progression of the disease. This outcome highlights that thiols could be an impressible biomarker for predicting of the severity of COVID-19.

## 1. Introduction

A new variant of coronavirus was found out at the end of the 2019 [1]. The infection brought on by the novel causative agent, severe acute respiratory syndrome coronavirus 2, was broadly accepted as coronavirus disease-2019 (COVID-19) [1,2]. COVID-19 has been notified from more than one hundred countries across the world, culminating in pandemic. The COVID-19 outbreak constitutes a notable challenge for individuals and society, as well as health systems and communities [2]. 

COVID-19 exhibits a wide array of clinical characterizations from asymptomatic illness to severe lung disease and/or multiorgan failure [3]. The effectiveness of the host’s immune response has a very substantial influence on establishing the clinical manifestations [3]. In the course of viral infection innate and adaptive immune cells get involved in immune response [4–7]. Studies have demonstrated that SARS-CoV-2 is able to effect both the respiratory epithelial cells and alveolar macrophages [4,5]. Moreover, SARS-CoV-2 has capability to replicate in lung tissues resulting in activation of innate immune response and augmentation of cytokines generation [5,6]. It is well revealed that SARS-CoV-2 triggers a Th1 type immune response and reduces circulating NK cells and T-cell subsets [3–7]. 

Inflammation is a crucial component of functional immune response [3,8]. It is hard to effectively eliminate infections in the absence of inflammation [3]. Although, SARS-CoV2 induces extreme cytokine responses in certain infected subjects, recognized as a cytokine storm [8,9]. It can be obviously stated that if there is a deficiency in the protective immune components including Th1 responses and cytotoxic CD8 cells, the immune dysregulation occurs [4]. Immune dysregulation results in a series of mechanisms that begin with acute inflammation, leading to cytokine storm, lung damage, and multiple organ failure [4,8,9].

Protein thiols and low molecular weight thiols like cysteine, glutathione form plasma thiols [10]. It is well known that thiols participate in cellular functions ranging from proliferation, apoptosis to inflammation and immune response [10,11]. Additionally, thiols are the main elements of antioxidant defense system [12]. A peptide thiol, glutathione (GSH), has a protective role in getting under control the proinflammatory situation in the lungs [13]. Nuclear factor kappa (NFKB) is a transcription factor that takes a significant part in the activation of inflammatory response [13,14]. Lung inflammation may stimulate the transcription factors like NFKB that regulates the expression of proinflammatory and antioxidant genes. GSH downregulates the activity of NFKB [13–15]. This indicates that the redox status of thiols may be a vital key in the modulation of the inflammatory responses in pulmonary tissue. 

Several reports have demonstrated the impact of thiol status in various infections and inflammatory diseases [16]. As thiol status is a good indicator of the cellular redox and have critical roles in several cellular signaling pathways, in this study, we aimed to investigate thiol levels in patients suffering with COVID-19. Until now, there has been no report regarding thiol status in SARS-CoV-2 infection, so this study constitutes the first paper in this area.

## 2. Materials and methods

### 2.1. Study design

For this prospective study, patients who were suffering from COVID-19 and admitted to Ankara City Hospital were included. Ankara City Hospital, which is responsible for the COVID-19 care assigned by the government, is one of the main hospitals in Turkey. The study procedure was set in accordance with the basis of the Helsinki Declaration and confirmed by the local ethics board (date: 11/11/2020, number: E1/1190/2020). Clinical diagnosis and classifications were performed in compliance with the directory of the World Health Organization (WHO) for COVID-19 and Diagnosis And Treatment Protocol for COVID-19 Patients (Trial Version 8). Confirmed cases having a positive RT-PCR detection of the novel coronavirus nucleic acid and/or imaging characteristics of COVID-19 were enrolled in the study. Patients having unconfirmed diagnosis of SARS-CoV-2 infection were excluded. Healthy individuals having a negative RT-PCR test results for SARS-CoV-2 represent the control group. A detailed history was taken from all participants and all subjects got examined extensively and had routine blood tests. Epidemiologic characteristics and radiologic imaging and routine laboratory test results were derived from electronic medical records. 

Venous blood samples were collected from each participant via venipuncture immediately on admission to the hospital within 24 h after diagnosis with SARS-CoV-2 infection. Afterward samples were processed by centrifugation at 1500 g from 10 min. Separated serum samples were frozen and held at –80 until the analysis was done. Serum thiol concentrations were determined with 5,-5’-dithiobis-(2-nitrobenzoic) acid (DTNB) according to the spectrophotometric assay defined by Erel & Neselioglu [10]. Measurements were made using a Siemens ADVIA 1800 chemistry analyzer (Siemens Healthcare GmbH, Erlangen, Germany).

### 2.2. Statistical analysis

Variables were evaluated regarding their distribution by both visual (histograms, plots) and statistical tests (the Kolmogorov–Smirnov test and the Shapiro–Wilk test). Continuous variables were represented as means and standard deviations or medians and interquartile ranges when appropriate in respect to their distribution. Categorical variables were expressed by numbers and percentages (%). To compare differences among groups a one-way ANOVA with a Bonferroni post-hoc test and a Kruskal–Wallis, along with a Dunn’s multiple comparison post-hoc test was used for analyses. Categorical variables were evaluated by the chi-square or Fisher’s exact test. Between-group comparisons (control group vs. patient group) were applied using Student’s t-test or Mann–Whitney U test. Correlation analyses were performed using Pearson’s correlation for normal distributions and the Spearman’s correlation test for nonnormal distributions. The optimal cut-off value, sensitivity, and specificity of thiol test were defined by analysis of receiver operating characteristic (ROC) curve. Multinomial logistic regression analysis was conducted to determine the independent risk factors for the severity of COVID-19. All the statistical calculations were performed using the Statistical Package for Social Sciences (SPSS) software program (v.22; IBM, Armonk, NY, USA) and a P value less than 0.05 was set statistically significant for all analyses.

## 3. Results

A total of 517 patients with confirmed SARS-CoV-2 infection were enrolled in the study, among them 200 (38.7%) were female and 317 (61.3%) were male. The patients were categorized into groups according to the severity of disease: 172 cases were severe group (90 as severe and 82 as critical), and 345 cases were non-severe group (85 as mild and 260 as moderate). The control group consisted of 70 healthy individuals (44 male/26 female). The mean age of the patient group was 63.81, and the mean age of the control group was 41.29. The most prevalent presenting symptoms were dyspnea, fever, and cough. Hypertension and chronic lung disease were the main underlying diseases followed by cardiovascular diseases and diabetes in severe and critical group. The demographic and clinical features of participants with COVID-19 were summarized in Table 1. 

**Table 1:** Clinical characteristics of patients with COVID-19.

	Mild COVID-19 casesn = 85	Moderate COVID-19 casesn = 260	Severe COVID-19 casesn = 90	Critical COVID-19 casesn = 82	P*
Age, mean ± SD	52.1 ± 14.16	63.34 ± 14.54	68.71 ± 13.48	71.23 ± 12.84	< 0.05
Sex					< 0.05
Male	41 (48.1)	157 (60.4)	64 (72.6)	55 (69.3)	
Female	44 (51.9)	103 (39.6)	26 (27.4)	27 (30.7)	
Signs and symptoms					
Fever	13 (16.5)	41 (15.8)	20 (23.8)	15 (20)	> 0.05
Cough	17 (21.5)	42 (16.2)	21 (25)	8 (10.7)	> 0.05
Dyspnea	11 (13.9)	63 (24.2)	17 (20.2)	25 (33.3)	<0.05
Fatigue	10 (12.7)	34 (13.1)	11 (13.1)	15 (20)	> 0.05
Nausea and vomiting	8 (10.1)	15 (13)	3 (3.6)	5 (6.7)	> 0.05
Myalgia, arthralgia	5 (6.3)	17 (6.5)	3 (3.6)	3 (4)	> 0.05
Diarrhea	3 (3.8)	8 (3.1)	1 (1.2)	-	> 0.05
Headache	3 (3.8)	5 (1.9)	2 (2.4)	-	> 0.05
Comorbidities					
Diabetes	13 (16.5)	38 (14.6)	17 (20.2)	13 (17.3)	> 0.05
Hypertension	15 (19)	50 (19.2)	28 (33.3)	25 (33.3)	< 0.05
Cardiovascular disease	4 (5.1)	20 (7.7)	18 (21.4)	17 (22.7)	< 0.05
Chronic lung disease	9 (11.4)	52 (20)	25 (29.7)	23 (30.6)	> 0.05
Malignancy	1 (1.3)	5 (1.9)	3 (3.6)	4 (5.3)	> 0.05
Chronic kidney disease	-	3 (1.9)	3 (3.6)	7 (9.3)	< 0.05
Neurologic disorders	2 (2.5)	12 (4.6)	10 (12)	5 (6.6)	> 0.05

As thiol test results and certain laboratory findings of the subjects were represented in Table 2 and Figure A; thiol concentrations were significantly different when compared synchronously in all 5 groups (one-way ANOVA, P < 0.001). When post-hoc testing was conducted it was found that thiol levels were significantly lower in all COVID-19 groups than healthy group (P < 0.001, for all). Moreover, patients in the critical COVID-19 group had the lowest thiol amounts than the other COVID-19 groups (P < 0.05, for all). When evaluated based on routine blood tests, lactate dehydrogenase (LDH) values were significantly higher in patients in critical group than in other groups (P < 0.05, for all). Patients had significantly higher D-dimer, procalcitonin (PCT), C-reactive protein (CRP), ferritin, troponin I, urea, creatinine and aspartate aminotransferase (AST), and international normalized ratio (INR) in critical COVID-19 group than in mild and moderate COVID-19 group and controls (P < 0.001, for all). However, the patients in severe group had lower D-dimer, PCT, CRP, ferritin, troponin I, urea, creatinine and AST levels; these differences were not statistically significant (P > 0.05, for all). In terms of lymphocyte (LYM) counts, patients in critical group had the lowest LYM counts in all study groups. Although patients had higher LYM counts in severe COVID-19 group than critical group, the differences was not significantly significant (P > 0.05). The thiol test was negatively correlated with D-dimer, troponin I, ferritin, LDH, CRP, LYM, INR, urea, and AST levels (r = –0.52, r = –0.50, r = –0.50, r = –0.49, r = –0.47, , r = –0.45, r = –0.41, r = –0.40, r = –0.37, respectively; P < 0.001, for all). 

**Figure:**
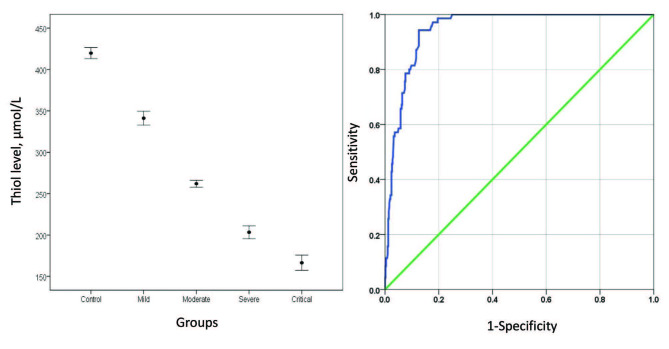
a. the alteration of thiol levels via the severity of COVID-19 (x̄   σx̅), b. ROC curve of the thiol test predicting COVID-19.

**Table 2:** Laboratory findings of individuals in study groups.

Parameters	Control group(n = 70)	Mild group (n = 85)	Moderate group(n = 260)	Severe group(n = 90)	Critical group(n = 82)	P value
Thiol, µmol/L	419.76 ± 55.88 b,c,d,e	341.13 ± 73.91a,c,d,e	262.02 ± 66.87 a,b,d,e	203.33±70.86 a,b,c,e	166.48 ± 79.4 a,b,c,d	< 0.001*
LDH, U/L	199 (45) b,c,d,e	244(130) a,c,d,e	292 (125) a,b,d,e	351 (147) a,b,c,e	443 (307) a,b,c,d	< 0.001
Troponin I, ng/L	2.5 (1.5) c,d,e	2.4 (0.6) c,d,e	4.74 (7.6) a,b,d,e	9.5 (28.4) a,b,c	19.5 (56) a,b,c	< 0.001
INR	1.00 (0.03) b,c,d,e	1.04 (0.11) a,c,d,e	1.09 (0.15) a,b,d,e	1.16 (0.16) a,b,c	1.17 (0.2) a,b,c	< 0.001
D-dimer, mg/L	0.315 (0.23) c,d,e	0.325 (0.30) c,d,e	0.790 (0.89) a,b,d,e	1.21 (1.56) a,b,c	1.230 (2.4) a,b,c	< 0.001
PCT, µg/L	0.03 (0.01) c,d,e	0.03 (0.03) c,d,e	0.04 (0.07) a,b,d,e	0.10 (0.17) a,b,c	0.19 (0.59) a,b,c	< 0.001
Ferritin, µg/L	38 (188) b,c,d,e	158 (233) a,c,d,e	288.5 (446) a,b,d,e	616.5 (695) a,b,c	674 (890) a,b,c	< 0.001
LYM, 109/L	1.90 (0.84) c,d,e	1.80 (0.82) c,d,e	1.03 (0.90) a,b,d,e	0.82 (0.45) a,b,c	0.60 (0.45) a,b,c	< 0.001
PLT, 109/L	261 (104)	269 (207) e	297 (191) e	265 (146)	234 (124) b,c	> 0.05
CRP, g/L	0.003 (0.006) c,d,e	0.006 (0.013) c,d,e	0.020 (0.044) a,b,d,e	0.040(0.08) a,b,c	0.075 (0.11) a,b,c	< 0.001
Urea, mg/dL	26 (9) b,c,d,e	34 (20) a,c,d,e	43 (27) a,b,e	52 (35) a,b	64 (66) a,b,c	< 0.001
CRE, mg/dL	0.72 (0.15) b,c,d,e	0.80 (0.29) a,e	0.87 (0.34) a,e	0.92 (0.57) a	1.03 (1.03) a,b,c	< 0.001
ALT, U/L	19 (17) b,c,d,e	44 (37) a	43 (42) a	36 (52) a	33 (27) a	< 0.001
AST U/L	16 (8) b,c,d,e	27 (19) a,d,e	29 (23) a,e	36 (29) a,b	49 (41) a,b,c	< 0.001

Receiver operating characteristic (ROC) analyses were performed to assess the value of the thiol test and other routine blood parameters to discern the patients with SARS-CoV-2 infection from healthy individuals. As shown in Figure B and Table 3, the area under curve (AUC) for the thiol test (0.949) was greater than the other parameters. The optimal cut-off level for thiol test was 323 µmol/L, the sensitivity was 0.986, and the specificity was 0.804. Multinomial logistic regression analysis was applied to identify the independent risk predictors for the severity of COVID-19. Thiol and ferritin levels were independent risk factors for the infection severity with each step of disease with the likelihood ratios presented in Table 4. Nevertheless, the risk of D-dimer and LYM were partial and other variables were unclear. Multivariate logistic regression analysis was performed to interpret the disease severity dependent variable using thiol and age independent variables. As a result of the analysis, a significant regression model was found [F (2.354) = 243.3, P < 0.001]. Overall, 58% of the variance in the dependent variable was explained by the independent variables. Accordingly, the independent variable thiol predicts the severity of the disease negatively and significantly (β = –0.61, t (354) = –13.52, P < 0.001, pr2 = 0.34). In addition, age, the independent variable, predicts the severity of the disease positively and significantly (β = 0.22, t (354) = 4.83, P < 0.001, pr2 = 0.062).

**Table 3:** The value of the studied parameters in predicting cases with COVID-19.

Variables	AUC (95% CI)	Std. error a	Asymptotic sig. b	Asymptotic 95% confidence interval
Thiol, µmol/L	0.949	0.009	< 0.001	0.931–0.968
Ferritin, µg/L	0.937	0.012	< 0.001	0.914–0.960
D-dimer, mg/L	0.829	0.023	< 0.001	0.784–0.874
LYM, 109/L	0.806	0.021	< 0.001	0.765–0.847
CRP, g/L	0.814	0.024	< 0.001	0.767–0.860
LDH, U/L	0.881	0.019	< 0.001	0.844–0.918
INR	0.843	0.022	< 0.001	0.801–0.885
PCT, µg/L	0.706	0.021	< 0.001	0.665–0.747
Troponin I, ng/L	0.739	0.025	< 0.001	0.691–0.787

**Table 4:** Multinomial logistic regression analysis.

Multinomial logistic regression models								
	Mild		Moderate		Severe		Critical	
Parameters	OR (95% Cl)	P value	OR (95% Cl)	P value	OR (95% Cl)	P value	OR (95% Cl)	P value
								
Thiol, µmol/L	0.975 (0.965–0.986)	< 0.001*	0.964 (0.953–0.976)	< 0.001*	0.953 (0.941–0.965)	< 0.001*	0.947 (0.935–0.960)	< 0.001*
Ferritin, µg/L	1.021 (1.011–1.030)	< 0.001*	1.021 (1.012–1.031)	< 0.001*	1.021 (1.012–1.030)	< 0.001*	1.021 (1.012–1.030)	< 0.001*
D-dimer, mg/L	NS	0.274	9.895 (2.274–43.051)	0.002*	10.054 (2.303–43.882)	0.002*	10.733 (2.455–46.868)	0.002*
LYM, 109/L	NS	0.794	NS	0.065	0.187 (0.06–0.535)	0.002*	0.062 (0.015–0.258)	< 0.001*
PLT, 109/L	NS	0.714	NS	0.922	NS	0.520	NS	0.074
LDH, U/L	NS	0.796	NS	0.807	NS	0.600	NS	0.105
Troponin I, ng/L	NS	0.177	NS	0.146	NS	0.131	NS	0.124
CRP, g/L	NS	0.419	NS	0.370	NS	0.259	NS	0.272
INR	NS	0.339	NS	0.311	NS	0.333	NS	0.402
PCT, µg/L	NS	0.038	NS	0.222	NS	0.541	NS	0.970

## 4. Discussion

The SARS-CoV-2 pandemic has expanded throughout the worldwide and generated a threat for societies, governments, and individuals [2]. COVID-19 displays a broad spectrum of clinical manifestations from asymptomatic to mild and moderate and eventually critical condition [3]. In the course of SARS-CoV-2 infection, replication of virus, immune and inflammatory responses are dynamic factors that can alter rapidly and lead various outcomes [4]. During viral infections both innate and adaptive systems concurrently take part in viral responses [7]. Production of several proinflammatory cytokines, activation of T cell and its subgroups are fundamental to take control the viral replication [6]. 

Thiols (-SH containing compounds) exist in protein motifs and function in enzyme modulation, signaling, inflammation, and immune response [10,11]. As the thiol redox system is vital, it changes in response to occurrence of disease, environmental factors, and several other conditions [16]. Alterations in the thiol network have pointed out a broad range of disorders. To the best of our knowledge, no prior report related to thiol status in COVID-19 has been published. 

Our results showed that thiol levels were lower in patients suffering from SARS-CoV-2 infection than controls. This data may suggest that thiols were used and depleted during the infection. There is a growing evidence that thiols have a crucial point in antioxidant defense, inflammation, and immune response [10–16]. Thus, thiols could be consumed during the SARS-CoV-2 infection which is related to a hyperinflammation status called a cytokine storm. The ROC analysis revealed that the thiol test provided an excellent discrimination between patients with COVID-19 and controls with an AUC of 0.949. Furthermore, it was observed that thiol levels decreased as the severity of the disease increased. In multinomial logistic regression analysis, thiols were found to be an independent risk factor for the severity of COVID-19. In addition, multivariate analysis revealed that the contribution of thiol in predicting the severity of the disease is considerably higher than age. These outcomes indicated that thiol status has a crucial position in the development and progression of COVID-19. Elevated CRP, PCT, and ferritin markers of inflammation were obtained in patients with COVID-19, when compared to the healthy controls. Also, the level of inflammation markers enhanced as the severity of the disease increases. This gradual increase of inflammation markers coincides with the gradual decrease of thiol levels. In addition, there were strong negative relationships between thiol concentrations and CRP, PCT and ferritin, markers of inflammation. Obtained data reflected that thiols are a notable key in inflammatory response to metabolic characteristics in SARS-CoV-2 infection. D-dimer levels that increased with the severity of the disease exhibited the activation of coagulation in severe and critical COVID-19. There were intense negative correlations between thiol amounts and D-dimer levels. Previous reports demonstrated that sulfhydryl groups on platelet surface have a role in platelet responses [17]. Platelets are known to have primary potential contributions in hypercoagulation and thrombotic events in COVID-19 [18,19]. This information suggests that there may be a relationship between depleted thiol levels and hypercoagulation in severe cases of SARS-CoV-2 infection.

It was recognized that the variability in the course of the disease is due to a disequilibrium between proinflammatory and antiinflammatory factors in the lung tissue [4]. Primarily, acute inflammation follows the respiratory tract infection. In certain individuals the immune system causes viral disruption and bring down the inflammation while the others produce cytokine storm and hyperinflammation that results in multiorgan injury [20]. Glutathione, which is a thiol containing tripeptide, modulates the angiotensin converting enzyme (ACE) expression and activity. The SARS-CoV-2 infected cells results in inhibition of ACE2 activity and diminished ACE2 expression [21]. ACE activity induces oxidative stress, inflammation, and apoptosis, whereas ACE2 leads antiinflammatory, antioxidative, and antiapoptotic effects. The imbalance between ACE and ACE2 activities ends in hyperinflammation and ARDS [21]. Thus, GSH and other thiols may provide a protective role against augmented inflammation in SARS-CoV-2 infection based on ACE-ACE2 system. The NF-κB (nuclear factor kappa B), signaling pathway is participated in the development of the conventional pathway of inflammation [22]. GSH inhibits the activation of NF-kB and therefore maintains the cytokine storm under control [21]. A recent study evaluated the effects of using glutathione in the 2 patients with COVID-19 [23]. Glutathione was found to be effective in remission of respiratory symptoms [23].

N-acetylcysteine (NAC) is both thiol and one of the components of GSH synthesis [21]. There have been several clinical studies examining the use of NAC in respiratory diseases [24,25]. NAC has been demonstrated to represent protective effects in ARDS [24]. Furthermore, NAC induces the of nuclear factor erythroid 2-related factor 2 (Nrf2), which modulates antioxidant enzyme genes and downregulates inflammation [24]. 

## 5. Conclusion

Thiol status is depleted in COVID-19. The decrease of the thiol levels reflects the gradual increase in the severity of SARS-CoV-2 infection. Intense associations between thiol amounts and inflammatory markers support the significant role of thiols in immune and inflammatory responses. These data underline that thiols can be a sensitive indicator for predicting the severity of COVID-19. Thiol based agents such as NAC, lipoic acid, and GSH, blocking the NF-kB signaling, are promising for the novel therapy approaches against cytokine storm and hyperinflammation in COVID-19.

## Funding

This research did not receive any specific grant from funding agencies in the public, commercial, or not-for-profit sectors.
